# End-of-life care in psychiatry: ‘one chance to get it right’

**DOI:** 10.1192/pb.bp.114.049684

**Published:** 2016-02

**Authors:** Nuwan Galappathie, Sobia Tamim Khan

**Affiliations:** 1 St Andrew's Healthcare, Birmingham

## Abstract

End-of-life care has been given increasing importance within healthcare settings. In June 2014, the Leadership Alliance for the Care of Dying People published *One Chance to Get it Right*. This nationally accepted guidance replaces previous end-of-life care pathways such as the Liverpool Care Pathway and outlines how dying patients should be managed irrespective of setting. Increasingly, patients with mental health problems are entering their final days of life within psychiatric in-patient or acute hospital settings, and psychiatrists need to be aware of the new guidance and ready to implement it within psychiatric practice.

In recent years there has been a range of strategies, guidance documents and evolving frameworks aimed at improving clinical practice within end-of-life care. This includes the *End of Life Care Strategy* by the Department of Health in 2008^1^ and the General Medical Council's (GMC's) guidance document *Good Practice in Decision Making*.^2^ These initiatives have led to the development of several end-of-life pathways such as the Gold Standards Framework (www.goldstandardsframework.org.uk) and the Liverpool Care Pathway.^3^ While these pathways have enabled improvements within areas such as diet, fluid and pain control, they have been criticised as being derived from attempts to translate hospice care to alternate settings and are therefore considered to be too generic, lacking a patient- and family-centred approach.^3^ In June 2014, the Leadership Alliance for the Care of Dying People (LACDP), a coalition of 21 national organisations including the Department of Health, the GMC, the Royal College of Physicians and the Care Quality Commission (CQC), published *One Chance to Get it Right*.^4^ This guidance replaces previous end-of-life care pathways and outlines how care for dying patients should be provided irrespective of setting. It highlights five priorities for care: recognition of anticipated death, sensitive communication, patient involvement, the needs of families and individual care plans (Box 1). The new guidance is applicable in England but adopts aspects of the Mental Capacity Act 2005 that are applicable to both England and Wales.

Approximately 500000 people die in England each year, and their end-of-life care pathways are changing, with 60% now dying in hospital or care home settings rather than their own homes. Less than 6% are transferred to specialist hospice care.^1^ Therefore, end-of-life care is likely to increasingly affect psychiatrists working in psychiatric in-patient settings and medical liaison posts. Further to previous literature on end-of-life care within psychiatric settings,^5-10^ the new guidance will require psychiatrists to update their knowledge and be ready to implement the new guidelines. We have reviewed the new guidance document and considered its implications for psychiatric practice.

**Box 1** Priorities for care of the dying person

‘Priority 1: This possibility [that a person may die within the next few days or hours] is recognised and communicated clearly, decisions made and actions taken in accordance with the person's needs and wishes, and these are regularly reviewed and decisions revised accordingly.

Priority 2: Sensitive communication takes place between staff and the dying person, and those identified as important to them.

Priority 3: The dying person, and those identified as important to them, are involved in decisions about treatment and care to the extent that the dying person wants.

Priority 4: The needs of families and others identified as important to the dying person are actively explored, respected and met as far as possible.

Priority 5: An individual plan of care, which includes food and drink, symptom control and psychological, social and spiritual support, is agreed, co-ordinated and delivered with compassion.’

Source: Leadership Alliance for the Care of Dying People.^4^

## Patient-centred care

The new guidance builds on pre-existing good practice but moves away from the process and rigidity of previous care pathways and instead refocuses on providing individualised care for the dying person to meet their needs and wishes. It essentially fosters a cultural change enabling care that is both flexible and focused on the needs of the person.

Patients who physically deteriorate, leading to concerns they might die, should be assessed by a doctor able to judge whether the condition is reversible or whether death is likely. We suggest that in psychiatric settings a referral for a specialist medical opinion would be required to decide this issue. For community patients this would be initiated by the patient's general practitioner (GP), whereas in in-patient psychiatric settings the primary care team or psychiatrist may need to initiate this referral. Where curative treatment is not possible, the situation must be sensitively explained to the patient in a manner appropriate to their circumstances.

It is important to recognise that rather than a specific diagnosis of ‘dying’, end-of-life should be considered as a gradual spectrum. Communication must be clear and specific. In particular, the patient must be sensitively informed that they are likely to die soon, unless they have indicated that they would not wish to know. Where appropriate, the clinician should explain when and how death might be expected and the basis for that judgement, while acknowledging and accepting any uncertainty about prognosis with an opportunity to ask questions.

### Communicating with patient and their family or carers

The patient and their family should be informed which doctor and nurse is responsible for their overall care. We suggest that for vulnerable psychiatric patients, efforts should be made to involve the family and those who can support the patient, such as chaplaincy. The guidance recommends that subsequent to being told the patient is likely to die, there should be ongoing, proactive, sensitive communication that is clear and addresses the patient's end-of-life needs. Communication should be respectful in tone and pace and undertaken in private settings. It should be two-way with staff listening to the needs of the patient and their family and addressing concerns as they arise. Understanding should be checked and documented. For psychiatric in-patients, we suggest the least restrictive principle should be followed and where appropriate, patients should be transferred to nursing home or hospice settings. However, transfer to such settings may not be possible, either because of risks related to the patient's mental disorder or lack of availability of beds. In addition, some long-term psychiatric in-patients may choose their ward as the place where they would like to reside during their final days. In these cases, the patient's wishes should be respected where possible and the psychiatrist would then need to take the lead in coordinating their end-of-life care. Within liaison psychiatry, there is a need to train and educate medical and surgical teams in identifying and treating common mental disorders, such as depression, that may arise during a terminal illness.^5^

### Capacity

In accordance with the Mental Capacity Act 2005, when a patient lacks capacity to receive information about dying or to make decisions regarding their end-of-life care, if there are no advance decisions applicable to the patient's circumstances or lasting powers of attorney in place, then the clinical team would need to make decisions on behalf of the patient as per their best interests. It should be noted that the time of writing it is not possible to make a valid advance decision refusing treatment in Northern Ireland.

In terms of decisions regarding palliative treatment, the patient's needs and wishes, as well as the wishes of their families and those identified as important to them, should be taken into account. When speaking with family or carers, staff should clearly explain whether they are consulting, informing or involving them in decision-making.

## Care planning

Individualised care plans must be developed and regularly updated to meet the patient's rapidly evolving needs. The timing of decision-making should also be carefully considered, for example decisions related to life-prolonging treatments should not be made by out-of-hours teams and should, where possible, be deferred until those concerned (e.g. the senior clinician and the patient's family or independent mental capacity advocate where appropriate) are available.

Patients must have individual care plans for food and fluids. Clinical teams must understand that these are basic human needs and should be met if the patient requests food or fluids, with assistance to feed being provided if required. Although patients can refuse food or fluids when offered, advance statements to refuse should not be considered to have effect.

Care plans should also cover symptom control such as pain management and include prompt referral to specialist palliative care teams. Such teams should be available for routine daytime support and should also provide out-of-hours telephone advice. We suggest that in in-patient settings specialist palliative advice is obtained to consider the most appropriate form of analgesia, for example intravenous syringe drivers may need to be replaced by oral morphine or fentanyl patches if appropriate. The *One Chance to Get it Right* guidance^4^ recommends that patients should have care plans that cover psychological, social, spiritual, cultural and religious needs. Sensitive empathic engagement with the patient is required to take a meaningful spiritual history.^6^ Care plans should be reviewed as the patient's condition changes and shared with those involved in the patient's care. There should be judicious use of medications for symptom control and in particular anticipatory medicines should target specific symptoms, have a clinical rationale for their introduction, be regularly reviewed and adjusted as needed for effect, and the reason for their use explained to the patient and their families. Psychiatrists working in in-patient or liaison settings should involve specialist palliative teams when prescribing such medications. Psychiatrists should also involve the patient's GP and palliative care team when considering do-not-resuscitate orders.

## Family and staff support

The time leading up to the death of a family member can engender a range of psychological, physical and emotional challenges. Psychiatrists should listen and acknowledge these concerns, providing support where possible. In psychiatric in-patient settings, efforts should be made to allow the family as much access as possible to visit the patient, including during their final hours. We suggest that in addition to family support, when psychiatric in-patients enter end-of-life care, the in-patient staff team will require a lot of emotional support which could be facilitated by members of the team itself, chaplaincy, the team psychologist, the visiting palliative care nurse or a trauma support counsellor. Opportunities for debriefing sessions as well as a hospital service with family support would also be helpful.

## Implementation

The new guidance document appears to demonstrate a good example of joined-up thinking. It is notably aligned with a range of other national standards including the National Institute for Health and Care Excellence (NICE) *Quality Standard for End of Life Care*,^11^ the GMC guidance on decision-making,^2^ Nursing and Midwifery Council's *Professional Code of Conduct*^12^ and General Pharmaceutical Council's *Standards of Conduct*.^13^ NICE will also take the guidance into account when developing its new clinical guidelines on the care of dying adults. Clearly, implementation of the guidance will require appropriate support and training and, within National Health Service (NHS) trusts, assistance may be available from the trust' development authority. In addition, Health Education England has begun work to initiate e-learning programmes to enhance the training and education of health and social care staff involved in delivering end-of-life care (www.e-lfh.org.uk/programmes.end-of-life-care). To ensure standards are being met, the guidance will inform the new-style CQC inspection of hospitals with end-of-life being a core service area to be inspected. We suggest that, at an organisational level, implementation of the new guidance should be made a clinical priority. This could be enabled by NHS trusts and independent organisations appointing clinical leads to oversee training and education within this field.

## Conclusions

Psychiatrists, especially those working in long-term in-patient settings and medical liaison posts, will need to demonstrate that they have taken on board and implemented the new end-of-life care guidance. There is a need to develop training for psychiatrists in this area.
